# Developing a Polygenic Risk Score for Weight Gain in People Treated for Psychosis – Application in a Real-World Setting

**DOI:** 10.1192/bjo.2026.11949

**Published:** 2026-06-30

**Authors:** Yasitha Illangasekera, Camila Marcelino Loureiro, Mark Shakespeare, Adam Jameson

**Affiliations:** 1 Salford Royal Hospital, Salford, United Kingdom; 2 Manchester University, Manchester, United Kingdom; 3 Sheffield Hallam University, Sheffield, United Kingdom; 4 University of Peradeniya, Kandy, Sri Lanka; 5 RDASH NHS Foundation Trust, Doncaster, United Kingdom; 6 University of Bradford, Bradford, United Kingdom

## Abstract

**Aims:**

Genetic factors are thought to play an important role in antipsychotic-induced weight gain (AIWG). Polygenic risk scores (PRS) could provide a measure of genetic predisposition to antipsychotic drug induced weight gain (AIWG). We conducted a study to examine how a PRS, generated using SNPs, identified from a recent meta-analysis, related to weight-change over time in people with first episode-psychosis (FEP).

**Methods:**

The PRS included SNPs in six different genes, identified as having significant associations (p<0.05) with AIWG. These were *HTR2C* rs3813929; *MTHFR* rs1801133; *ADRA2A* rs1800544; *MC4R* rs489693; *LEPR* rs1137101 and *CNR1* rs1049353*.* An additive PRS and a risk allele based weighted PRS were created based on risk allele counts and presence or absence of risk alleles respectively. The additive PRS was also used to create low/high genetic risk groups for analysis. The association between PRS and weight gain per day (WGPD) in grams/day as well as BMI percentage change (=>7%) was investigated using regression models.

**Results:**

In multiple regression analysis, the additive PRS significantly predicted AIWG in females (adjusted r²=0.59, B: unstandardised regression coefficient=24.4 grams/day p <0.05), but not in males. ANCOVA showed that high genetic risk groups had greater WGPD (p=0.018), with significant PRS gender interactions driven by markedly higher WGPD in high-risk females (p=0.039).

Follow-up comparisons indicated that in females, those with ≥7 risk alleles had substantially higher WGPD scores (adjusted Mean=138.8 grams/day, 95% CI [99.6, 178.1]) compared with those with ≤6 alleles (adjusted Mean=40.4 grams/day, 95% CI [−5.5, 86.2]). In males, WGPD scores were not significantly different across genetic risk categories.

**Conclusion:**

We report a PRS that is predictive of weight gain in women treated for first episode psychosis, accounting for 59% of the variance daily weight-gain over time. Validation of the PRS in an independent cohort is required, as is determining whether it is feasible to apply the PRS prospectively in real world clinical settings to inform lifestyle measures and pharmacotherapeutic decisions in the treatment of FEP

‘et al’

Dr Adrian Phillipson, Sheffield Hallam University

Prof. Gavin P. Reynolds, Sheffield Hallam University

Prof. Caroline Dalton, Sheffield Hallam University

